# Unexpected Properties of Degassed Solutions

**DOI:** 10.1021/acs.jpcb.0c05001

**Published:** 2020-08-05

**Authors:** Barry W. Ninham, Pierandrea Lo Nostro

**Affiliations:** † Department of Applied Mathematics, Research School of Physics, Australian National University, Canberra, Australian Capital Territory 0200, Australia; ‡ Department of Chemistry “Ugo Schiff” and CSGI, University of Florence, 50019 Sesto Fiorentino, Firenze, Italy

## Abstract

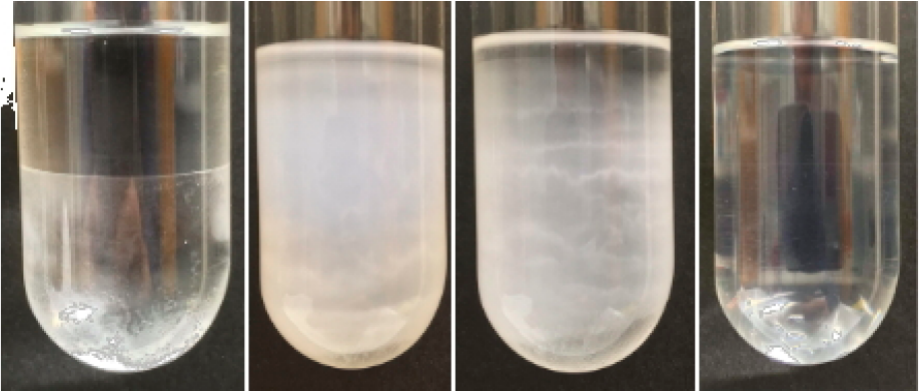

Theories of liquids and their simulation ignore any physical effects of dissolved atmospheric gas. Solubilities appear far too low to matter. Long-standing observations to the contrary, like cavitation, the salt dependence of bubble–bubble interactions, and the stability of degassed emulsions, continue to call that assumption into question, and these questions multiply. We herein explore more unexpected effects of dissolved gas that are inexplicable by classical theory. Electrical conductivities of different salts in water were measured as a function of concentration before and after degassing the liquid. The liquid/liquid phase separation of binary mixtures containing water, *n*-hexane, or perfluorooctane was significantly retarded after degassing. We anticipate that preliminary attempts at explaining these effect probably lie in self-organization of dissolved gas, like nanobubbles and cooperativity in gas molecular interactions. These are salt- and liquid-dependent.

## Introduction

It is a trite observation that if gases did not dissolve in liquids, life could not exist. The low solubilities of O_2_ and N_2_ are 6.352 and 11.92 mL/kg at 20 °C from moist air at 1 atm total pressure, respectively.^1^ Gases are considered either as inert molecular guests or ignored. At the same time, there is awareness that there may be a state of self-organization of gases in liquids, *nanobubbles*, about which there is much debate. Some examples of little understood effects of gases in liquids are O_2_, N_2_, and CO_2_ exchange in aquatic environments, in mammals and insects, in the use of O_2_-saturated perfluorocarbons as fluids for perfusion of transplant organs, and other degradable species. The radical-initiated peroxidation of lipids, the corrosion of metals, cavitation-induced damage in the propellers of ships, enzyme action, sonochemistry, and free radicals generally are indicative of the importance of the issue.^2^ The effects are obvious, but their origins are very obscure. The existence, stability, size, distribution, and reactivity properties of (nano)bubbles in water and organic liquids have been investigated extensively in bulk solution and at surfaces,^3,4^ but the important issues that such experiments intersect with have remained elusive.^5^ Some extraordinary phenomena remain unexplained. Perhaps the simplest is that of bubble–bubble coalescence in water.^6^ This is either strongly inhibited or quite unaffected by different salts. The inhibition occurs around the same critical ionic strength (0.17 M) for one class of salts. Significantly, this is precisely physiological concentration.^7^ For other salts, there is no effect. There are simple rules that determine which ion pairs induce the effects.^8^

Similar systematic effects occur with neutral solutes like sugars.^6^ Degassing changes the Hofmeister series for the flocculation rates of colloids^9^ and enhances the stability of emulsions.^10,11^ Gas-effected changes in solution properties are typified by two examples: (i) the cloud point of a short-chain phospholipid (dioctanoyl-phosphatidylcholine) in water dispersion and (ii) the formation of a polypseudorotaxane obtained from a poly(ethylene glycol) and α-cyclodextrin. In the first case, degassing brought about an increment in the cloud point temperature (*T*_cp_) from 23.4 to 24.9 °C, and the readmittance of a gas resulted in the lowering of *T*_cp_, depending on the nature of the gas.^12^ In the second experiment, degassing promotes the formation of polypseudorotaxanes, with a significant acceleration of the kinetics.^13^

We report here on further, and extraordinary, effects of degassing that bear on these issues: (1) For aqueous electrolytes, the electrical conductivity drops or increases depending on the specific ion pair and its concentration. The results are parallel and suggest insights into the bubble–bubble inhibition phenomenon linked to nanobubbles. (2) The phase separations of a water/hydrocarbon, water/fluorocarbon, and hydrocarbon/fluorocarbon binary solutions are significantly affected by the removal of the dissolved gases. This reinforces earlier hints that at least some “hydrophobic interactions” are strongly dependent on dissolved gas.

## Methods

We used deionized purified Milli-Q water from Millipore with a resistivity of 18.2 MΩ and a conductivity of 0.055 S/cm. *n*-Hexane (C_6_H_14_, 95% pure) was purchased from Sigma-Aldrich-Fluka (Milan, Italy), and perfluorooctane (C_8_F_18_, 98% pure) was purchased from Fluorochem Ltd. (Glossop, UK). No further treatments were carried out to improve the purity of these materials. KF, KCl, KBr, KI, KSCN, KClO_3_, and CH_3_COOK were purchased from Sigma-Aldrich-Fluka and were desiccated overnight in the oven at 353 K. All liquids were degassed in high vacuum test tubes of different capacities purchased by Disa s.a.s. (Sesto San Giovanni, Italy).

### Degassing Method

In a freeze–pump–thaw procedure, the liquid is first frozen with liquid nitrogen liquid, and then a vacuum of about 0.1 Pa is applied. The sample is excluded from the vacuum line and left standing at room temperature until the liquid thaws. This process was repeated four times.

### Electrical Conductivity

Electrical conductivity measurements were carried with a sensIon+ EC7 from HACH Lange (Lainate, Italy) equipped with a universal conductivity cell and an automatic temperature compensation. The output conductivity values were converted at 298 K. Because of the large difference in conductivity between pure water and salt solutions, two different probe models were used, i.e., sensIon+ 50 70 and sensIon+ 50 71.

### Light Transmittance

**Figure 1:**
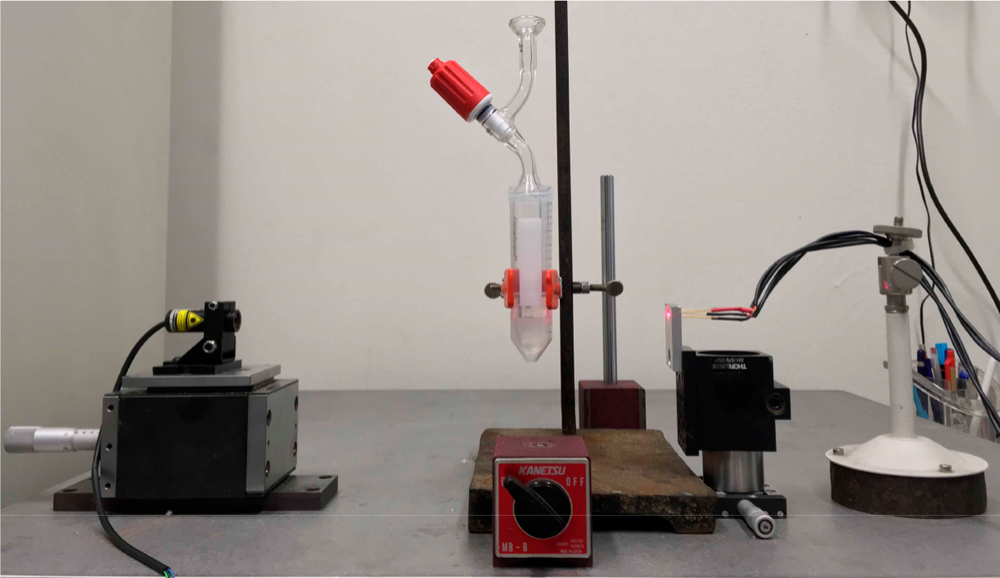
Setup for light transmittance measurements. By moving the vial up or down, the laser is allowed to go through the upper or the lower phase.

The instrument used for light-transmittance measurements was custom-built by assembling CW laser diode modules and a SPOT series segmented photodiode, both purchased from RS Components Italia (Milan, Italy). The experimental setup is shown in Figure 1. The laser has a nominal emission wavelength of 670 nm. The segmented photodiode has four separate active areas, and its spectral response ranges between 350 and 1100 nm. The laser is connected to a 6 V power supply, while the four quadrants of the photodiode are connected to four voltage inputs of a National Instruments DAQ board to display and record the experimental data.

### Phase separation

The samples for phase separation were prepared by adding in the high vacuum test tubes the proper amounts of water + *n*-C_6_H_14_ (sample A), water + C_8_F_18_ (sample B), and *n*-C_6_H_14_ + C_8_F_18_ (sample C) with a volume fraction of ϕ = 0.5. Each sample was vigorously shaken for 10 s, and then light transmission was acquired under three different conditions: standard (the sample is in equilibrium with the atmosphere), after degassing, and after re-exposure to air (regassing). Sample C was heated up to 333 K in order to completely mix the two liquids, then left to slowly cool down to room temperature while recording the light transmission.

## Results and Discussion

### Effects on Conductivity

Electrical conductivity measurements of water and solutions of KF, KCl, KBr, KI, KSCN, KClO_3_, and CH_3_COOK were performed at 25 °C at different concentrations (*c*), before and after degassing (see Table 1).

**Table 1:** Specific Conductivity (κ, in mS/cm) of Aqueous Solutions of Salt at 25° C at Different Concentrations in the Presence of Dissolved Gases and after Degassing^a^

		κ (mS/cm)											
		0.5 M			0.15 M			0.05 M			0.005 M		
pair	salt	gassed	degassed	Δ	gassed	degassed	Δ	gassed	degassed	Δ	gassed	degassed	Δ
α/α	KF	45.7	45.2	–0.6	14.62	14.63	0.01	5.79	5.83	0.04	0.602	0.607	5 × 10^–3^
	KCl	54.2	53.6	–0.6	16.69	16.65	–0.04	7.03	7.08	0.05	0.762	0.767	5 × 10^–3^
	KBr	59.6	59.1	–0.5	18.59	18.55	–0.04	7.10	7.13	0.03	0.771	0.776	5 × 10^–3^
	KI	60.5	60.0	–0.5	19.81	19.78	–0.03	7.21	7.24	0.03	0.770	0.774	4 × 10^–3^
α/β	KClO_3_	56.6	56.7	0.1	17.00	17.01	0.01	6.15	6.21	0.06	0.708	0.714	6 × 10^–3^
	CH_3_COOK	41.5	41.8	0.3	13.05	13.11	0.06	5.00	5.04	0.04	0.569	0.575	6 × 10^–3^
	KSCN	57.2	57.4	0.2	18.05	18.06	0.01	6.42	6.47	0.05	0.718	0.723	5 × 10^–3^

a Δ indicates the difference in κ between the degassed and the gassed states.

The results show the following: (i) The difference in conductivity after degassing the solution is small but reproducible, with a good standard deviation. (ii) The salts behave practically in the same manner when their concentration is 0.005 and 0.05 M, that is, below 0.17 M. At such low concentrations, we expect that the conductivity would be predicted from a theory involving only electrostatic interactions in a *continuum* water background. (iii) Instead, the salts behave quite differently and specifically when their concentration is 0.5 M. This concentration is greater than the critical concentration of 0.17 M, beyond which bubble–bubble fusion inhibition occurs for that ion pair.

To summarize these results more explicitly: For the bubble–bubble interaction problem, the coalescence behavior is determined not by the cation or the anion but by the particular *combination* of ions present. The rule that determines behavior, that is, the on/off inhibition or no inhibition, is that cations and anions are assigned a property or “flavor”, α or β. The rules are as follows: α/α and β/β pairs stop bubble fusion, and α/β and β/α pairs have no effect on bubble fusion. Here we observe the following: (a) The α/α pairs show reproducible lowering in the measured conductivity when degassed. (b) The α/β pairs show instead a smaller increment in the measured conductivity when degassed.

In summary, when the salt concentration is lower than the “magic” 0.17 M, the degassing does not significantly affect the conductivity. Only slight positive changes can be recorded (i.e., degassing increases the conductivity a little bit). When the salt concentration is greater than 0.17 M then the conductivity changes significantly, more than an order of magnitude larger with respect to the dilute regime. The key observation is that the conductivity effects correlate with and depend on the α/α or α/β pairs. We remark here that certainly in a moderately concentrated salt solution (0.5 M) nonelectrostatic interactions dominate; the Debye screening length is about 0.5 nm.

**Figure 2:**
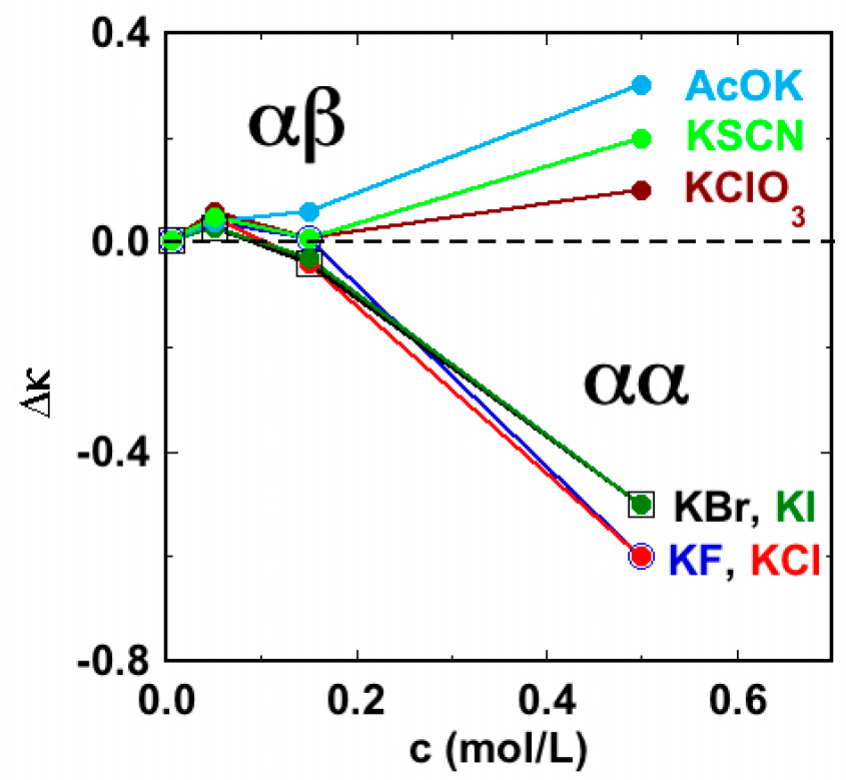
Conductivity change (degassed – gassed, mS/cm) as a function of the salt concentration.

Potassium halides inhibit bubble–bubble coalescence and are classified as α/α salts. However, potassium acetate is an α/β salt and does not affect the phenomenon.^6^ Similarly, KClO_3_ and KSCN are α/β electrolytes and behave like potassium acetate, as depicted in Figure 2 where the conductivity change between the degassed and the gassed solutions is plotted as a function of the salt concentration.

The results indicate that, as in the bubble–bubble coalescence study, when *c* < 0.17 M all 1:1 salts behave in the same way; in fact, κ increases after degassing regardless of the composition of the salt. However, when *c* > 0.17 M, κ drops for α/α salts after degassing, but it remains unaffected in the case of α/β electrolytes, increasing with potassium acetate.

The phenomenon cannot be simply explained in terms of the Hofmeister series. In fact KBr and KI, which are classical chaotropic species, do behave like KF and KCl that possess a kosmotropic nature. Furthermore, the concentration threshold of about 0.17 M is more or less the same as that found in the bubble–bubble coalescence studies, the same salt concentration as that for the blood of all animals.^7^

### Effects on Liquid/Liquid Phase Separation

**Figure 3:**
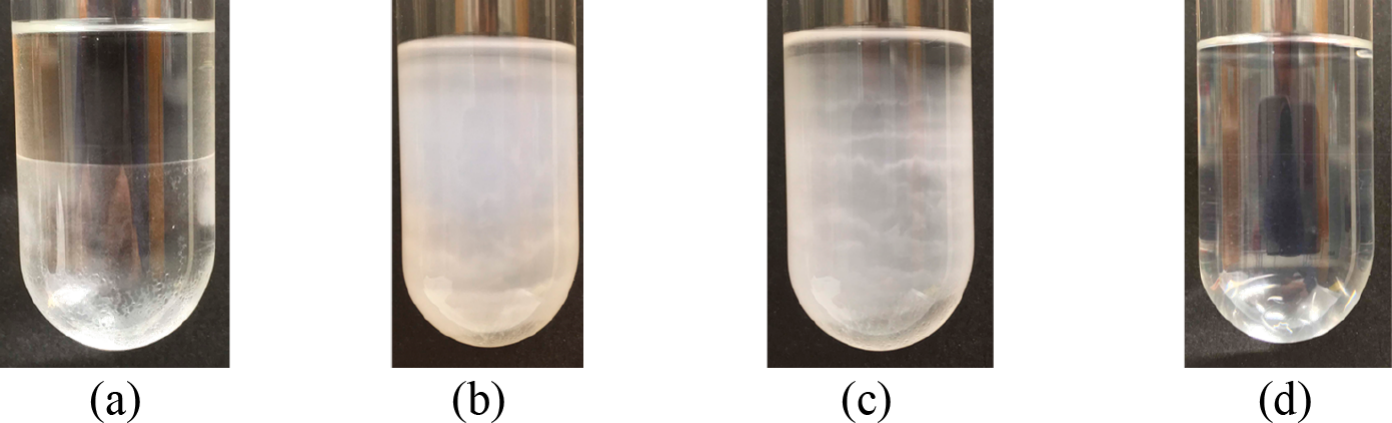
Time-lapse photographs of a C_8_F_18_/*n*-C_6_H_14_ mixture (sample C). (a) *t* = 0, (b) *t* = 60 s, (c) *t* = 360 s, and (d) *t* = 1020 s.

An equally unexpected effect occurs with emulsions of oil and water. Upon degassing, demixing is strongly inhibited. For oils of the same density as water, the emulsions are indefinitely stable. These observations of Pashley defy the accepted canons of physical chemistry as much as the salt dependence of bubble–bubble interactions.^10^ They imply and confirm something radical but long suspected: that “hydrophobic interactions ” do not exist without dissolved gas! We have carried these observations further by studying the remarkable changes in the kinetics of demixing when water is shaken with other immiscible liquids such as a hydrocarbon (HC) or a fluorocarbon (FC). Under normal conditions, the gravity-driven macroscopic demixing occurs in few seconds. If the liquids are degassed and shaken, then they remain mixed for up to some hours before they separate. Figure 3 shows the evolution of a degassed *n*-C_6_H_14_/C_8_F_18_ system set at rest, after vigorous shaking, up to 17 min.

The Pashley phenomenon had been shown to occur in water/hydrocarbon and water/perfluorocarbon systems.^11^ Like the bubble–bubble inhibition phenomenon, the emulsion stability problem was simply disbelieved and dismissed as being due to impurities, the usual panacea for inconvenient truths.

We have further extended the study to a water-free hydrocarbon/fluorocarbon system. Depending on the chain length and on the concentration, HC/FC mixtures phase separate below a certain temperature *T*_c_, a result of their mutual structural incompatibility.^14^ For example, for the perfluorooctane/*n*-hexane system, *T*_c_ = 314.0 K when the mole fraction of FC is about 0.26.^15^ The concentration of dissolved gases is much higher in hydrocarbons and fluorocarbons than in water. For instance, the solubility of O_2_ in water, *n*-C_8_H_18_, and C_8_F_18_ at ambient pressure and temperature is approximately 31, 288, and 521 mL/L, respectively. Interestingly, the gas dissolving capacity decreases with the molecular volume (or polarizability) of the solute in the order CO_2_ ≫ O_2_ > CO > N_2_ > H_2_ > He.^16^

We found a consistent and reproducible delay in the time required for complete macroscopic separation of the two liquids, compared to the same system shaken in normal conditions. The results on the behavior of the single different samples are given below.

#### Water/*n*-Hexane

**Figure 4:**
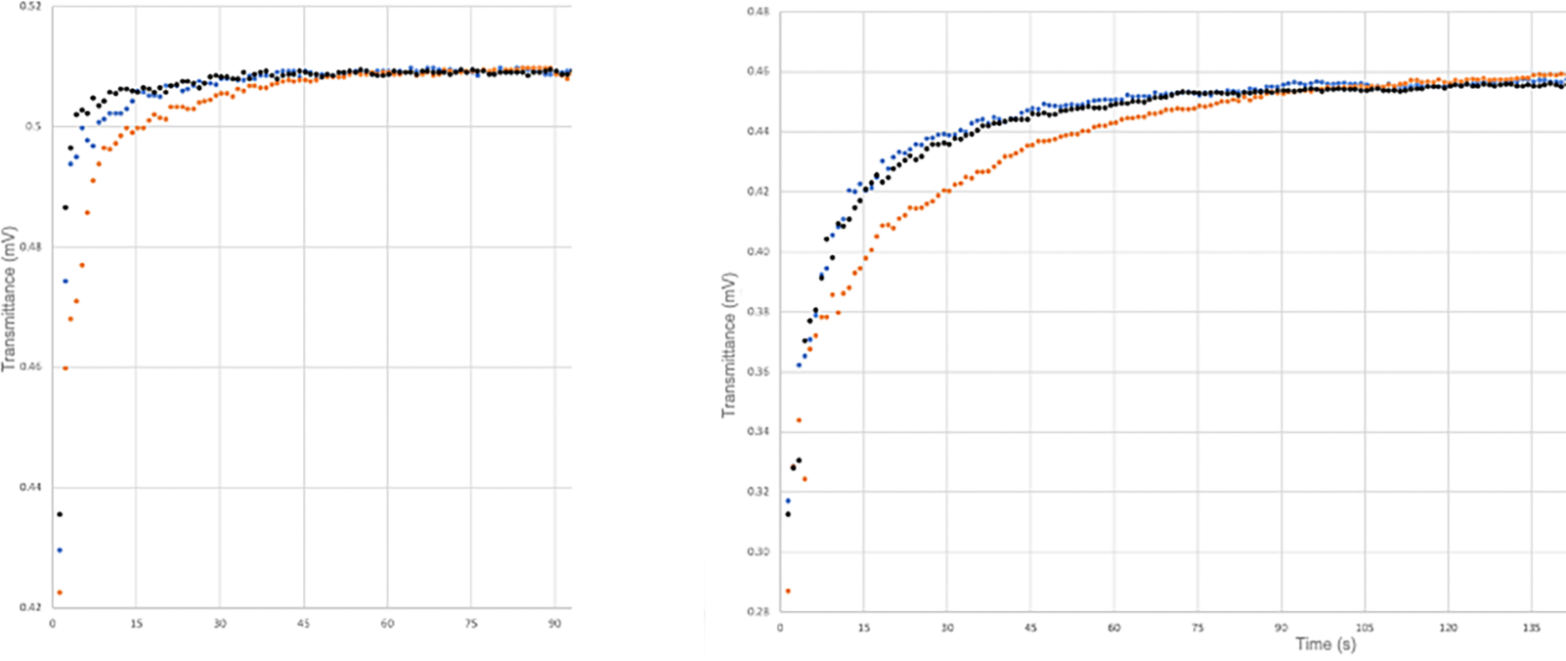
Light transmittance (left: across the upper, *n*-hexane phase; right: across the lower, water phase) during the phase separation of *n*-C_6_F_14_/water (sample A). Blue: standard condition, red: after degassing, black: after regassing.

Figure 4 shows the variation of light transmittance across the upper phase (*n*-hexane) as a function of time and indicates that upon degassing (squares) the phase separation is at least three times slower than in normal (circles) or regassed (triangles) conditions. The phenomenon is fully reversible.

#### Water/Perfluorooctane

**Figure 5:**
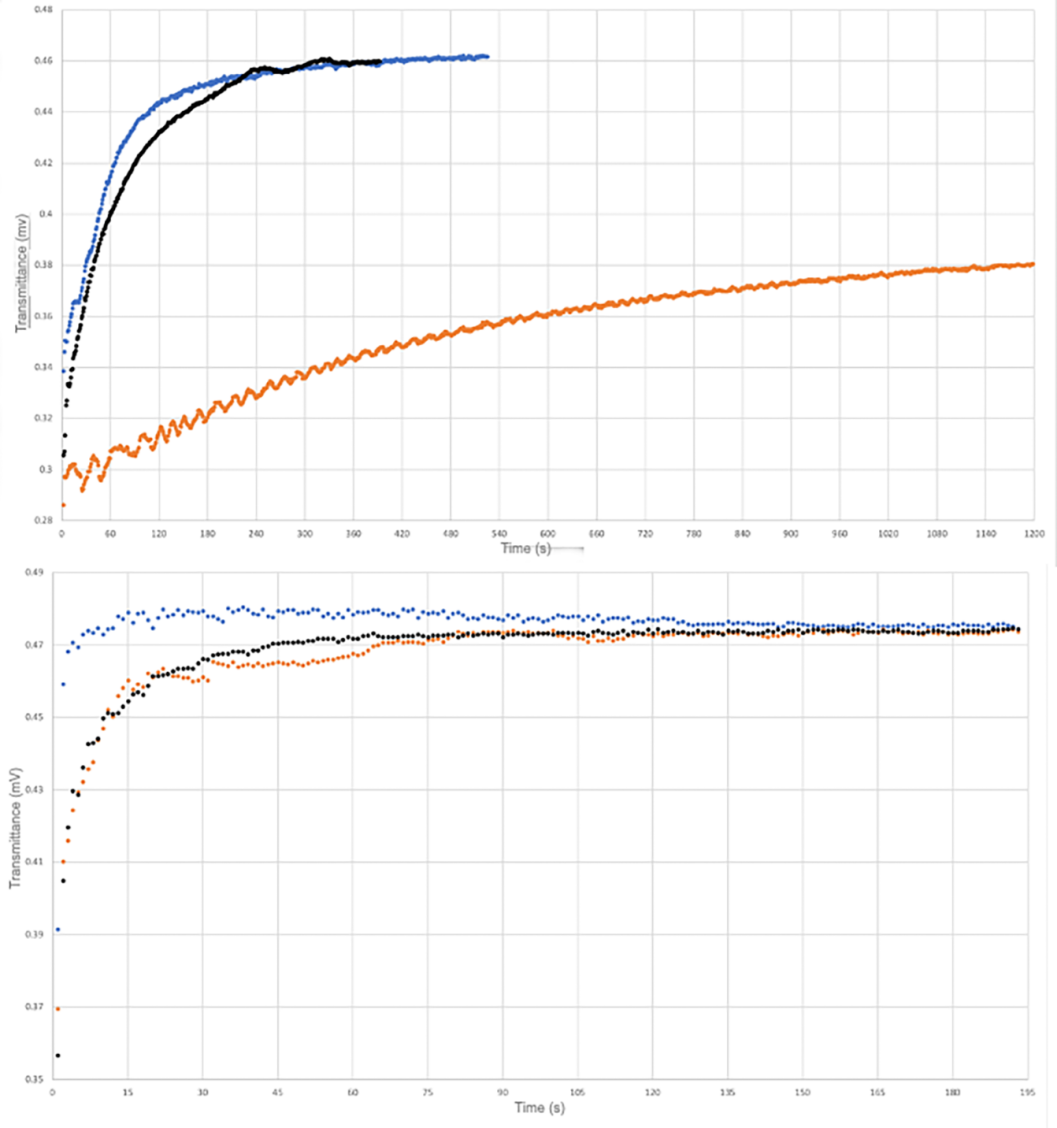
Light transmittance (top: across the upper, water phase; bottom: across the lower, fluorocarbon phase) during the phase separation of water/C_8_F_18_ (sample B). Blue: standard condition, red: after degassing, black: after regassing.

Figure 5 shows the variation of light transmittance across the upper phase (water) and the lower (fluorocarbon) phase as a function of time. Interestingly, the interphase between the two liquids is a relatively stable opaque emulsion that takes time to break. Degassing has a huge effect on the stability of the dispersion. After 20 min, the oil was still dispersed, and this is consistent with the results obtained by Pashley during a study of degassed perfluorohexane in water dispersions.^17^ Furthermore, the degassed water/perfluorooctane sample shows the onset of oscillations in light transmitted across the upper phase (red curve). The comparison between the regassed curve (in black) and the regular profile (in blue) shows that the phenomenon is not fully reversible. The bottom plot in Figure 5 reports the light transmittance across the lower (C_8_F_18_) phase during the separation. It indicates two interesting effects. The stability of the water dispersion in perfluorooctane is very similar to that of water in hexane. This means that the phase separation occurs in a short time, i.e., the removal of water from perfluorooctane is much faster than that of the fluorocarbon from the aqueous layer. This indicates that the thick intermediate emulsion is richer in water. Again, the phenomenon is not readily reversible, as the red and black curves overlap.

#### *n*-Hexane/Perfluoroctane

**Figure 6:**
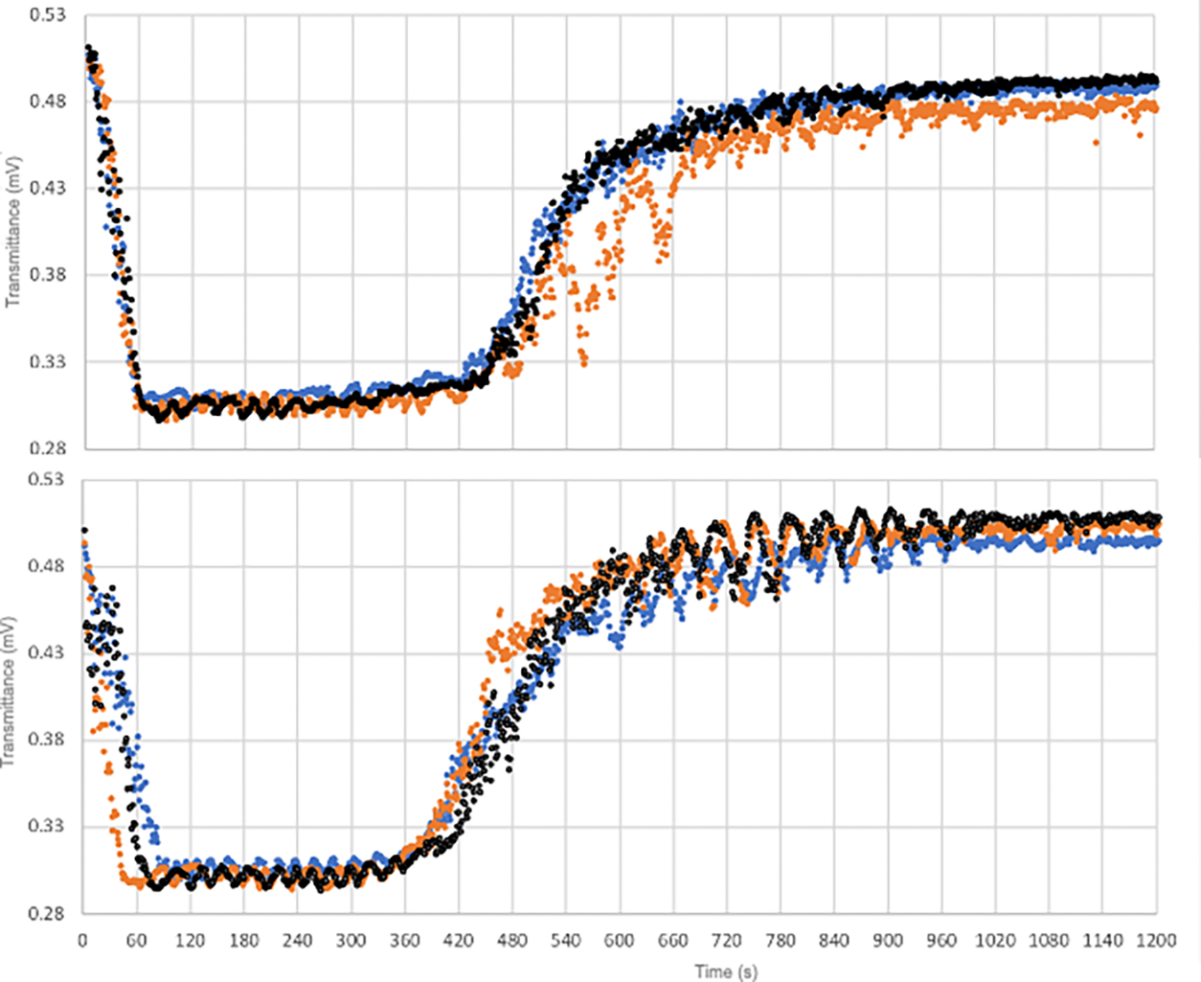
Light transmittance (top: across the upper, *n*-hexane phase; bottom: across the lower, fluorocarbon phase) during the phase separation of *n*-C_6_H_14_/C_8_F_18_ (sample C). Blue: standard condition, orange: after degassing, black: after regassing.

The phase separation process can be divided in three steps that are reflected in the pictures shown in Figure 6. When the temperature of the mixture drops below the upper consolute temperature,^15^ the system begins to separate, and in 1 min, the minimum in light transmittance is reached.

Instead, after degassing for about four minutes the separation of *n*-hexane and perfluorooctane seems to be inhibited. This inertia could be explained by the little difference in the internal pressure (∂*U*/∂*V*)_*T*_ between the two organic compounds. This parameter reflects the strength of the intermolecular interactions that in the case of perfluorooctane and *n*-hexane are pretty weak (mainly London forces). A closer look at pictures (b) and (c) in Figure 3 suggests that the phase separation is more evident at the bottom and at the top of the tubes, where pure C_8_F_18_ and *n*-C_6_H_14_ are accumulating, because of the large density difference between the two liquids (1.7567 and 0.6545 g/mL, respectively).^15^

The laser and sample holder were aligned in such a way to acquire the data at least 0.5 cm above and below the interphase as shown in Figure 1. For this reason, the spectra acquired (see Figure 6) are similar to each other for the first six minutes. After this time, when the metastable intermediate phase gets thinner, the light transmission increases, and the difference between the profiles of the two layers increases remarkably.

Figure 6 (top and bottom) shows the variation of light transmittance across the upper phase (*n*-hexane) and the lower (fluorocarbon) phase as a function of time. Interestingly, although *n*-hexane and perfluoroctane dissolve more gases than water, the degassing (orange profile) does not affect the separation time; instead, it significantly affects the frequency and amplitude of the oscillations.

In particular, for the *n*-hexane upper layer the frequency and the amplitude of oscillations in the intermediate region increases between 500 and 700 s, i.e., the time frame when the laser irradiates the interphase between the liquids, and this suggests that the removal of gases mostly affects mostly the evolution of the interface. This phenomenon occurs also in the perfluorooctane lower phase (bottom panel in Figure 6).

The readmittance of atmospheric gases (regassing) confirms what we already observed for sample B: In the *n*-hexane phase, the process is reversible, while in perfluorooctane it is not. However, the effect of hexane in the presence of perfluorooctane is weaker than that in the presence of water. This intriguing result suggests that the effect of degassing in the phase separation kinetics is not limited to systems containing water.

In summary, we have reported solid experimental evidence for a remarkable effect of dissolved gases in two different cases, the conductivity of strong electrolytes, and the phase separation of two immiscible or partly miscible liquids. We have also outlined the different effect induced by electrolytes on the conductivity of their aqueous solutions in terms of their propensity to promote or inhibit bubble coalescence, and we have shown that the effect of dissolved gases in the liquid/liquid phase separation process is not limited to water but appears to be a general phenomenon that involves any liquid.

The bubble–bubble fusion inhibition problem is about the simplest experiment one could imagine. It has been known and was used for coal flotation for over a hundred years. In passing, we recall that they used sea water, where the salt concentration reaches about 0.6 M, enough to make nanobubbles stable. These adsorb at the particles’ surface and cause flotation. It has been systematically quantified for 40 years.^6,18^ This is obviously a key issue in physiology where the blood concentration has an effective ionic strength of precisely the magic 0.17 M.^19^ This is the concentration of the ocean in the Permian era when land animals first emerged.

Classical DLVO theory is utterly unable to explain the phenomenon.^20^ “Impurities” are not an answer. Mechanisms involving the molar surface tension increment of the specific electrolyte, the Gibbs elasticity, the adsorption, or the ion partitioning have been proposed.^8,21^ None are successful.

We propose that the answer may lie in the following hypothesis: After much debate about the existence and stability of nanobubbles at surfaces and in bulk, there is general agreement that nanobubbles can exist as stable entities in high salt.^3,22^ Whatever arcane arguments exist as *pro* and *con*, the fact is that they do exist and are crucial to our own existence.^7^

At around 0.17 M salt, there is a critical nanobubble formation concentration. The nanobubbles adsorb cations and bind anions just as do ionic micelles.^23−25^ Anion binding is highly specific and falls into two classes. For example, for quaternary ammonium surfactants, binding of Br^–^ and Cl^–^ is 80–90%. For acetate or OH^–^ or ClO_3_^–^, the critical micelle concentrations (CMCs) are much higher, and there is no counterion binding.^26^

For the liquid/liquid phase separation, we argue that the gas nanobubbles promote the phase separation between the two immiscible liquids through interfacial adsorption at the outer surface of oil droplets in water. This means that phase separation is promoted by buoyancy. The removal of gases inhibits this gravitational effect and the oil becomes more stable in water. Moreover, the adsorption of ions, especially chaotropic species, at the water/nanobubble interface can promote phase separation through electrostatic repulsion, but the great solubility of gases in perfluorocarbons may suggest that states of self-organization of dissolved gases such as nanobubbles are at the core of the mechanism that produces the observed effects with degassing.

The forces between two molecularly smooth surfaces charged by adsorbed surfactants are measured in a solution of the surfactant our macrobubbles. Above the CMC, it is only the “unbound” counterions that contribute to the electrostatic forces between them.^27^ These are also augmented by depletion forces due to micellar size,^28^ so the double-layer forces become strongly repulsive above the CMC.^27^

Now, if we replace mica surface, micelle, and CMC by the words macrobubble, nanobubble, and “critical nanobubble concentration” (CNC), then we have explained our phenomenon. The recorded change in conductivity reflects and supports the postulated critical nanobubble concentration.

This accommodates our α/α and β/β ion pairs. For the others, α/β and β/α with little or no binding, there is no CNC, and the forces and conductivity are normal electrostatics and weaken, screened with increasing salt.

There is a very large number of phenomena to which this explanation may apply, including the growth of inorganic particles,^29,30^ pH and buffer effects on polyelectrolytes and proteins,^31^ specific ion effects,^32^ and more.

Another possible contribution that can participate in the phenomena we have been studying is the preferential adsorption of OH^–^ ions at the air/water interface of nanobubbles that brings about a corresponding increase in the proton concentration in the bulk.^33^ This phenomenon should imply a lowering in conductivity when the solution of an α/α electrolyte that is supposed to stabilize nanobubbles is degassed. The opposite result is expected for a solution of α/β electrolytes that instead promote the coalescence of bubbles.

An accurate estimate of these effects on the conductivity of ionic solutions is not straightforward, in part because the ion-product constant of water (*K*_w_) is expected to change upon degassing.^33^ However, a preliminary, approximate calculation gives a result in line with the proposed mechanism and the shift in the isoelectric point at the surface of water.^34^

Furthermore, Figure 2 shows that at high concentrations (0.5 M) each salt has a specific behavior, particularly in the case of the α/β pairs. This observation should imply that the interfacial adsorption of OH^–^ ions does not fully account for the observed changes, but there must be a specific contribution to the variation of the conductivity. This effect is shown also by the α/α pairs, with an interesting difference between cosmotropic (F^–^ and Cl^–^) and chaotropic (Br^–^ and I^–^) ions.
